# A Neural Framework for Age-Related Multi-Omics Discovery

**DOI:** 10.1093/geroni/igaf122.2237

**Published:** 2025-12-31

**Authors:** Bartek Nogal, John Earls, Nathan Price

**Affiliations:** The Buck Institute, Navato, California, United States; The Buck Institute, Navato, California, United States; The Buck Institute, Navato, California, United States

## Abstract

Aging is a dynamic, multifactorial, and nonlinear process, shaped by genetics, environment, and stochastic forces. Polygenic risk scores (PRS) provide a powerful framework, with advancing genomic technologies capturing greater trait variance and enhancing their utility in aging research. Nonetheless, many studies rely on linear analytical approaches that can overlook the complex and variable nature of aging biology. GWAS-based phenotypes often mask sub-phenotypes, limiting the granularity of PRS-driven insights. Also, they don’t account for when in the life course genetic risk manifests into disease, and what aging-related mechanism makes the vulnerability arise. A single age-related PRS, for instance, may align with multiple omics features in deeply phenotyped cohorts, which can propel biomarker discovery. Yet, relying on linear methods, PRS often explain relatively modest variance and can be confounded by aging—losing predictive power once age is incorporated. To address these challenges, we leverage a perturbable variational autoencoder (VAE) trained on nearly 2,000 individuals with over 100 PRS and 1,200 multi-omics features, explicitly modeling age-related effects. This unsupervised neural architecture captures complex relationships, enabling exploration of nonlinear aging trajectories. We uncovered both established and potentially novel associations, including metabolic and mitochondrial pathways in Alzheimer’s disease, lysosomal processes in Parkinson’s disease, and steroid sulfation in frailty and longevity phenotypes. To contextualize these findings, we integrated a Retrieval-Augmented Generation (RAG) system, coupling large language models with structured database queries. By combining PRS, omics-based modeling, and LLM-driven interpretation within a perturbable VAE framework, we enable robust inference and advance mechanistic discovery of aging-related traits.

